# Rearing systems alter growth performance, behavior, brain characteristics, and meat quality in slow-growing Korat broiler chickens

**DOI:** 10.1016/j.psj.2026.106916

**Published:** 2026-04-09

**Authors:** Pramin Kaewsatuan, Wichuta Khosinklang, Sirirot Khotsalee, Catleya Rojviriya, Wunpen Sonsamrong, Mutyarsih Oryza, Amonrat Molee, Wittawat Molee

**Affiliations:** a School of Animal Technology and Innovation, Institute of Agricultural Technology, Suranaree University of Technology, Nakhon Ratchasima 30000, Thailand; b Synchrotron Light Research Institute (Public Organization), Nakhon Ratchasima 30000, Thailand

**Keywords:** Slow-growing chickens, Rearing systems, Behavior, Brain porosity, Meat quality

## Abstract

Alternative rearing systems have been increasingly adopted to improve poultry welfare and product quality. This study investigated the effects of organic **(OR)**, free-range **(FR)**, and conventional **(CO)** rearing systems on growth performance, behavior, brain characteristics, fatty acid profiles, and meat quality of slow-growing Korat chickens **(KRC)**. A total of 375 mixed-sex day-old chicks were randomly allocated to 15 pens (5 replicates per treatment) and reared until 91 days of age. All birds were housed indoors at 5 birds/m², while FR and OR groups were provided access to Ruzi pasture at 1 and 4 m²/bird, respectively, from day 21. Behavioral activities were recorded by video observation during days 84 to 91. At slaughter, feather condition, brain structure, and meat quality were evaluated. Results indicated that OR chickens had significantly lower feed intake and final body weight compared with CO and FR birds (*P* < 0.05). Conversely, CO birds exhibited poorer feather condition, particularly in the tail region (*P* < 0.05). Principal Component Analysis **(PCA)** revealed distinct behavioral patterns among rearing systems, with CO birds displaying higher frequencies of aggressive behaviors (pecking, fighting), whereas FR and OR birds showed greater expression of nutritive and resting behaviors. Chickens reared under FR and OR systems exhibited lower brain porosity and greater brain volume compared with CO birds (*P* < 0.05). Regarding meat quality, FR and OR systems increased collagen content and shear force, while FR meat showed the lowest drip loss (*P* < 0.05). Moreover, the OR system significantly increased total n-3 polyunsaturated fatty acid (**PUFA**) content in breast meat (*P* < 0.05). Collectively, these results indicate that environmental complexity shapes brain integrity and behavioral resilience in KRC. This supports the adoption of welfare-oriented management practices, such as pasture access, to reduce aggression and improve meat quality. Such an approach may facilitate premium product differentiation, enabling producers to offset the economic constraints associated with slower growth rates through added welfare and nutritional value.

## Introduction

In recent years, the global demand for poultry meat has surged, driven by population growth and the need for affordable protein sources (Magdelaine et al., 2008). Concurrently, there has been a paradigm shift in consumer preferences, with an increasing emphasis on food safety, natural products, and high animal welfare standards (Vanhonacker and Verbeke, 2009; Vanhonacker et al., 2016). To address these demands while ensuring food security and sustainable production amidst these evolving market trends, the livestock sector is increasingly focusing on ethical farming practices that align with the fundamental principles of sustainable production (Gamage et al., 2023). Consequently, the adoption of alternative rearing systems, such as free-range **(FR)** and organic **(OR)** farming for slow-growing breeds, constitutes a strategic approach to ethically and sustainably bolstering dietary protein supply while enhancing the competitiveness of small- and medium-scale farmers in developing regions.

High stocking densities in conventional systems (20–24 birds/m²) are known to induce chronic stress (Ravindran et al., 2006) and trigger adverse physiological responses (Marin et al., 2001). Conversely, extensive systems like FR and OR prioritize animal well-being (Mikulski et al., 2011). Guided by European Commission Regulations (EC No 889/2008; EC No 543/2008), which mandate slow-growing genotypes and continuous outdoor access, these systems encourage natural behaviors such as foraging, dust bathing, and exercise (Rasmussen et al., 2024).

This study focuses on the Korat broiler chicken (**KRC**), a slow-growing crossbreed derived from male Leung Hang Khao (**LHK**), an indigenous Thai breed, and female Suranaree University of Technology (**SUT**) synthetic line, with a growth rate of 19.8-21.0 g/d and a market weight of approximately 1.2 kg at 65 days (Kubota et al., 2019). Due to its high adaptability, heat tolerance, and disease resistance, the KRC is highly compatible with extensive farming systems. These traits, combined with its favorable meat texture and flavor (Katemala et al., 2021), have established the KRC as a strategic genotype aimed at enhancing the sustainability of local poultry production and meeting the growing demand for premium poultry meat.

Literature regarding the influence of rearing systems on performance and meat quality remains inconsistent. While some studies report comparable growth between FR and indoor birds (Jiang et al., 2011; Mikulski et al., 2011), others suggest FR birds exhibit lower weight gain and feed efficiency (Castellini et al., 2002; Wang et al., 2009). Likewise, findings on tenderness and nutritional value vary, with reports of FR meat being superior (Jiang et al., 2011; Sun et al., 2012), inferior (Castellini et al., 2002; Mikulski et al., 2011), or unchanged (Wang et al., 2009; Chen et al., 2013). A notable exception is meat color; chickens raised in FR (Castellini et al., 2002; Fanatico et al., 2007; Sales, 2014) and OR (Küçükyilmaz et al., 2012; Molee et al., 2022) consistently showed increased yellow pigmentation due to carotenoid intake. Additionally, rearing systems influence fatty acid profiles and flavor, while promoting the accumulation of zinc and iron through diverse foraging and exercise-induced myogenesis (Castellini et al., 2002; Overbeke et al., 2006; Husak et al., 2008; Lin et al., 2014). Zinc and iron are fundamentally involved in muscle metabolism and oxidative stability (Pearce et al., 2009; Duffy et al., 2023); therefore, achieving higher concentrations of iron and zinc is likely to enhance the overall nutritional density and quality of KRC meat.

Moreover, several studies have confirmed that conventionally confined systems often compromise bird welfare by inducing stress through high stocking densities (Bessei, 2018). Mechanistically, this stress triggers the activation of the hypothalamic–pituitary–adrenal (HPA) axis, which has been identified as a key factor driving adverse behaviors such as feather pecking (De Haas et al., 2014). In contrast, access to range areas has been shown to alleviate such damage. This mitigation is likely due to the diverse environment and increased space for exercise, which allows birds to engage in natural behaviors rather than redirected pecking (Rodriguez-Aurrekoetxea and Estevez, 2016). Beyond behavioral expression, recent evidence suggests that the environmental complexity in free-range systems also stimulates brain plasticity, particularly in the hippocampal complex and the nidopallium caudolaterale—regions vital for spatial cognition and stress regulation (Tahamtani et al., 2015). However, to date, limited research has simultaneously investigated these brain structural adaptations in slow-growing chickens reared under extensive systems (Mehlhorn and Petow, 2020). Specifically, how the KRC neurobiologically adapts to these environments remains largely unexplored.

Therefore, the present study aimed to investigate growth performance, behavioral expression, brain structure (specifically porosity and volume), fatty acid profiles, and meat quality of slow-growing chickens across OR, FR, and CO systems. We hypothesized that birds raised in alternative systems (FR and OR) would exhibit superior welfare status—reflected by neuroanatomical integrity and natural behaviors—as well as enhanced performance and meat quality compared to those reared in conventional indoor systems. The present study provides novel insights into the neurobiological and physiological adaptations of KRC to different rearing environments. By providing a scientific basis for welfare-oriented management, these findings empower producers enhance animal welfare and meat quality, supporting product differentiation in an increasingly competitive market.

## Material and methods

### Ethics statement

All experimental protocols and procedures used in this research were reviewed and approved by the Ethics Committee on Animal Use of the Suranaree University of Technology. Nakhon Ratchasima, Thailand (SUT-IACUC-010/2021).

### Birds, experimental design, diets, and measured parameters

The trial was conducted at the Poultry Research Unit of SUT Farm, located at latitude 14°53′13″ N and longitude 101°59′42″ E from June to September 2021. Throughout the experiment, the mean daily temperature ranged from 20°C to 35°C with an average RH of 76% (Nakhon Ratchasima Meteorological Department, Nakhon Ratchasima, Thailand), and the photoperiod was 16 h of light and 8 h of darkness. The heavy metal content in water and soil was measured, with no detectable levels observed. Additionally, the pasture had not been exposed to pesticides or herbicides for three years before the start of organic production.

A total of 375 one-day-old mixed-sex Korat broiler chickens were used as experimental animals and reared until slaughter age (day 91). Vaccinations were administered to all birds to protect them from Marek’s disease on day 1, Newcastle disease, and infectious bronchitis on day 7 and day 21. The Gumboro disease vaccine was given on day 14. No beak trimming or other pharmacological treatments were performed on all birds. All birds were randomly allocated to fifteen pens assigned to three rearing systems—control (**CO**), free-range (**FR**), and organic (**OR**)—with five replicates per treatment and 25 birds per replicate, following a completely randomized design. The OR birds were kept in a house in accordance with the Thai Agricultural Standard: Organic Agriculture Part 2—Organic Livestock (TAS 9000 Part 2-2018).

In the CO group, birds were maintained in open-sided indoor pens with rice husk litter (10 cm depth) at a stocking density of 5 birds/m². These pens were equipped with side curtains and fans for regulated ventilation and cooling. While birds in the FR and OR groups were initially housed under the same indoor conditions, they were provided free access to outdoor pasture areas starting from day 21 until slaughter at stocking densities of 1 and 4 m²/bird, respectively. The pasture area was planted with Ruzi grass from seed and grown by irrigation. Throughout the day, the FR and OR groups of birds could freely access both indoor and outdoor areas, but they were confined to indoor pens at night. Feed and water were provided inside the housing with hand-filled feeders and nipple drinkers, respectively, to allow *ad libitum* intake. The chickens were given 3 mixed feeds throughout the experiment: starter from days 1–21, grower from days 22–42, and finisher from days 43–91. The same experimental feed mixture was provided to all groups to ensure consistent nutrient and ingredient compositions across the three systems (Table 1). Due to the lack of established breed-specific nutrient requirements for the Korat broiler chickens, diets were formulated based on the NRC (1994) recommendations. In accordance with regulations, the organic feed mixture contained at least 90% organically grown ingredients, with the remainder derived from approved conventional sources. The chemical composition of experimental feeds was determined according to standard procedures (AOAC, 2010) and presented in Table 1.

**Table 1:** Compositions and calculated nutrient contents of the trial diet.

Feed ingredients	Starter (1-21 days)	Grower (22-42 days)	Finisher (43-91 days)
Full fat Soybean meal	47.70	41.00	34.50
Broken rice	48.50	55.65	62.35
DL-methionine	0.25	0.10	0.10
Salt	0.35	0.35	0.35
CaCO_3_	1.40	1.30	1.20
Monocalcium phosphate (21% P)	1.30	1.10	1.00
Premix^1^	0.50	0.50	0.50
**Calculated nutrients (%)**			
ME (kcal/kg)	3,175	3,190	3,200
Crude protein	21.00	19.00	17.00
Crude fat	9.60	8.60	8.00
Crude fiber	2.73	2.37	2.05
Digestible Lysine	1.21	1.08	0.95
Digestible methionine	0.59	0.43	0.41
Digestible Met + Cys	0.93	0.73	0.69
Calcium	1.00	0.90	0.85
Available phosphorus	0.45	0.38	0.35

1 Premix (0.5%) provided the following per kilogram of diet: 15,000 IU of vitamin A; 3,000 IU of vitamin D3; 25 IU of vitamin E; 5 mg of vitamin K3; 2 mg of vitamin B1; 7 mg of vitamin B2; 4 mg of vitamin B6; 25 µg of vitamin B12; 11.04 mg of pantothenic acid; 35 mg of nicotinic acid; 1 mg of folic acid; 15 µg of biotin; 250 mg of choline chloride; 1.6 mg of Cu; 60 mg of Mn; 45 mg of Zn; 80 mg of Fe; 0.4 mg of I; 0.15 mg of Se.

Weekly measurements of bird and feed weights were conducted to determine body weight (**BW**), body weight gain (**BWG**), feed intake (**FI**), and feed efficiency in each pen. All calculations for BWG and feed efficiency were adjusted for mortality. The feed conversion ratio (**FCR**) was calculated using the recorded total BWG and FI data:

$$FCR=\frac{FI}{BWG}$$

### Assessments of pecking damages and behaviors

Physical welfare indicators were assessed on all birds present in each pen when they reached market weight (day 91). Plumage damage, serving as evidence of injurious pecking, was scored individually for the back, thighs, wings, and tail of each bird using a 6-point scale adapted from Phillips and Heins (2021). The scoring criteria were as follows: 0 = fully feathered, 1 = rough, 2 = some broken feathers, 3 = heavily broken feathers, 4 = almost bald, and 5 = bald.

Manual behavioral analysis was performed on chickens aged 84 to 91 days, representing the final stage of the production cycle where birds have fully adapted to their respective rearing systems, thus allowing for the observation of established behavioral patterns and the cumulative effects of environmental factors. Video recordings were collected during two daily observation periods: a morning session (09:00–12:00) and an afternoon session (13:00–17:00). Cameras were centrally positioned over the indoor pen and the 'front' and 'middle' sections of the outdoor range to ensure the birds were visible at all times. The observed behaviors included moving (e.g., foraging), eating (e.g., drinking, feeding), resting (e.g., sitting), comfort (e.g., preening, dust bathing, flapping wings, shaking, scratching, and stretching), and other behaviors (e.g., pecking, fighting) according to the protocol proposed by Bateson and Martin (2021). Total duration and bout number of all behaviors listed in the ethogram described previously (Chen et al., 2019), with a small modification, were recorded.

### Sample collection and chemical analysis

At day 91, a total of 20 birds from each treatment, comprising two males and two females from each of the 5 replicates, were randomly selected and slaughtered in the SUT slaughterhouse 12 h after feed fasting. All birds were electrically stunned, bled, mechanically de-feathered after scalding, manually eviscerated, and then stored at 4°C for 24 h until required for further analysis. The breast muscle from the left side of the pectoralis major muscle was used for muscle quality assessments, including physical characteristics, mineral content, and fatty acid profile. The portion of breast meat samples from the right side of the pectoralis major muscle were rapidly snap-frozen using liquid nitrogen and subsequently stored at −80°C for biochemical compound analysis.

Measurements of muscle pH and color were performed following the procedures of Wattanachant et al. (2004). A portable pH meter (−99163; Hanna Instruments, Inc., UK) was used to measure the pH of the breast muscle at 45 min and 24 h postmortem. Each pH value was calculated as the mean of three readings taken from separate points on the same sample. The color of the breast muscle and skin was determined at three distinct points using an automatic colorimeter (Chroma Meter CR-300; Minolta, Japan). Measurements of lightness (L*), redness (a*), and yellowness (b*) were averaged for statistical analysis.

The drip loss of the breast meat was determined following the bag method described by Honikel (1998). After chilling for 24 h, breast meat samples with a length, width, and height of 3.0 cm × 1.5 cm × 0.5 cm were excised from a consistent location on the muscle and weighed. Each sample was suspended in an individually labeled, airtight plastic bag to prevent contact with the bag surface and stored at a 4°C freezer for 24 h. Thereafter, the samples were removed from the bags, gently blotted to remove excess moisture, and reweighed. The percentage of drip loss was estimated by using the following formula:

$$Drip\;Loss\;\left(\%\right)=\;\frac{\left(Weight\;before\;storage-Weight\;after\;storage\right)}{Weight\;before\;storage}\times \;100$$

For cooking loss, the samples were prepared and processed according to the standardized procedures of Honikel (1998). Briefly, a breast meat sample was cut into pieces (1.5 cm × 3.0 cm × 0.5 cm), weighed, placed in sealed plastic bags, and cooked in a water bath at 80°C until they reached an internal temperature of 71°C. The samples were subsequently removed from the bags, gently blotted with paper towels to remove excess moisture, left to cool to room temperature, and reweighed to calculate the cooking loss percentage using the following formula:

$$Cooking\;Loss\;\left(\%\right)=\;\frac{\left(Weight\;before\;cooking-Weight\;after\;cooking\right)}{Weight\;before\;cooking}\times \;100$$

The shear force of the breast muscle samples was measured using a texture analyzer (TA-XT2, Texture Technologies Corp., Scarsdale, NY, USA). After thawing the samples overnight, each breast muscle was cooked in a water bath to an internal temperature of 80°C. Once cooled to room temperature, the cooked samples were cut into pieces with dimensions of approximately 2.0 cm in width, 3.0 cm in thickness, and 0.5 cm in length. The texture analyzer was set to a constant crosshead speed of 20 cm/min, and the maximum force required to shear through each sample was recorded as Warner-Bratzler shear force (WBS), expressed in kgf/0.5 cm². Shear force values were averaged from six measurements per sample, and the final shear force was calculated according to the method described by Wattanachant et al. (2004).

The lipid fraction for fatty acid evaluation was extracted from the breast meat following the method of Folch et al. (1957) using a chloroform-methanol (2:1, v/v) solution. The extracted lipids were then trans-esterified into fatty acid methyl esters (**FAMEs**) by methylation, following the method outlined by Metcalfe et al. (1966). The FAMEs were separated and analyzed using a gas chromatograph (Hewlett-Packard 6890 Series GC System, Agilent Technologies, Santa Clara, CA, USA) equipped with a flame ionization detector (**FID**) and a capillary column CP-Sil 88 (100 m x 250 µm i.d., 0.2 µm film thickness). Helium was the carrier gas with a 0.95 mL/min flow rate. The injector and detector temperatures were set at 260°C and 260°C, respectively, with the oven temperature initially programmed to rise from 70°C to 175°C at a rate of 13°C/min, then further increased to 240°C at a rate of 4°C/min. Fatty acid peaks were identified by comparing the retention times of the sample FAMEs with those of known standards (Sigma-Aldrich, St. Louis, MO, USA). The fatty acid composition was expressed as a percentage of the total identified fatty acids.

### Iron and zinc determination

The concentrations of iron and zinc were determined with slight modifications to the method described by Gerber et al. (2009). Briefly, thawed breast muscle samples (0.5 g) were digested by adding 4 mL of nitric acid (65% HNO_3_, suprapur, Merck, Darmstadt, Germany) and 1 mL of hydrogen peroxide (30% H_2_O_2_, proanalysis, Merck, Darmstadt, Germany). Automated Microwave Digestion System Discover® SP-D 80 was used for the digestion process. The mixture was heated at 150–200°C until the solution became clear. After cooling, the digested solution was diluted with deionized water to a final volume of 25 mL. Iron and zinc concentrations were then determined using an atomic absorption spectrometer. The spectrometer was set to a wavelength of 213.9 nm for zinc and 248.3 nm for iron. The absorbance of the digested sample solutions was measured and compared against the calibration curve to quantify the mineral content.

### Analyses—brain volume and porosity

Sixty birds (4 birds per pen; 2 males and 2 females; 20 birds per treatment) were randomly selected and euthanized under inhalation anesthesia using the volatile anesthetic isoflurane (Piramal Critical Care, Inc., Bethlehem, PA, USA) in a well-ventilated area. Once deep anesthesia was confirmed, the brain was immediately dissected by decapitation and subsequent craniotomy, and each tissue was weighed. The brain tissue was then rinsed with phosphate-buffered saline (**PBS**) to remove residual blood, preserved in a cryoprotectant solution using the method of Aurelia and Fahy (2015), and stored at −25 °C for subsequent analysis.

**Fig. 1:**
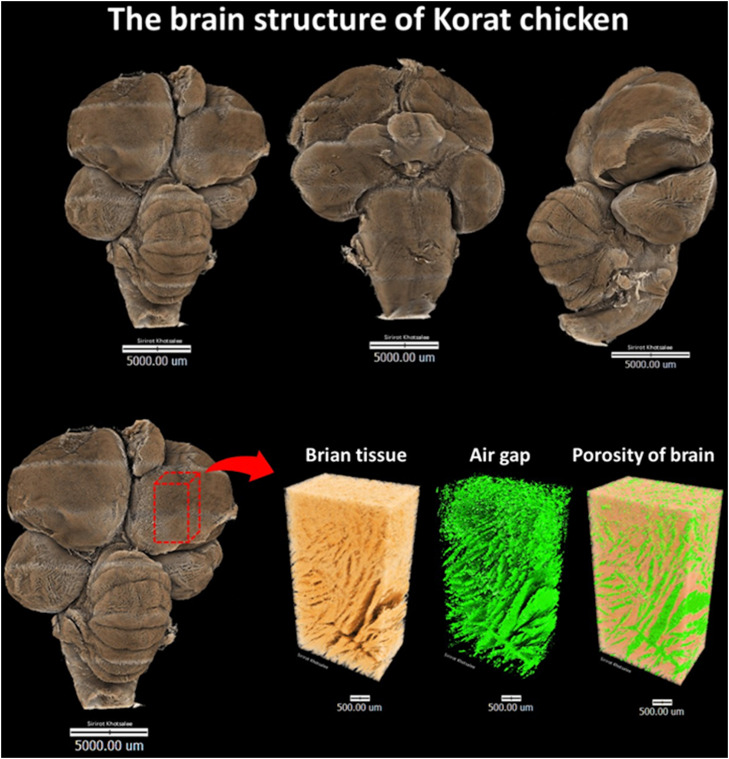
Illustrates the structural organization and porosity analysis of the Korat broiler chicken brain.

For the analysis, the brain samples were air-dried overnight before the SRXTM experiment. All XTM data were acquired using a Synchrotron radiation X-ray tomographic microscopy (SRXTM) beamline (BL 1.3 W) at the Synchrotron Light Research Institute (SLRI), Nakhon Ratchasima, Thailand. A full dataset of X-ray projections was acquired for each specimen, spanning a 180° rotation with a 0.1° angular step. To minimize the detrimental effects of scattering and beam hardening, the polychromatic X-ray beam was filtered with 400 µm of aluminum foil. This attenuation ensured that the X-ray energy utilized for imaging was maintained above 7 keV. X-ray images were recorded with a CMOS camera coupled to a LuAG scintillator detector, yielding an effective pixel size of 0.7 microns. The exact acquisition parameters were fine-tuned by the facility's technical experts to ensure optimal data quality for each sample. Fig. 1 illustrates the structural organization and porosity analysis of the Korat broiler chicken brain.

For data processing, the raw XTM data were first preprocessed and then reconstructed into three-dimensional volumes using the Octopus Reconstruction software, which implements a filtered-back projection algorithm. Following reconstruction, 3D reconstruction was performed using Octopus software to quantify porosity and volume of the brain (Vlassenbroeck et al., 2006).

### Statistical analysis

All statistical analyses were conducted using SPSS 29.0 software (SPSS Inc., Chicago, IL, USA). A one-way ANOVA within the GLM procedure was used to evaluate the effects of the rearing system on performance, meat quality, fatty acid composition, mineral content, and brain volume and porosity. Data are presented as mean and standard error of the mean (SEM), with significance set at *P* < 0.05.

For non-parametric behavioral traits and plumage condition, the Kruskal-Wallis test was applied. Data are presented as mean rank, with significance defined as *P* < 0.05.

Principal component analysis (PCA) of all behaviors was used to assess the differences and similarities across the three rearing systems using the R package i.e., FactoMineR (Lê et al., 2008) and factoextra R package (Kassambara and Mundt, 2017). After the bout number of behaviors was scaled (normalized), the principal component (PC) scores and loadings were estimated, and the PCA was performed using a correlation matrix.

## Results and discussion

### The effect of rearing systems on production performance

The production performance of KRC reared under three different systems is shown in Table 2. No mortality or clinical signs of illness were observed throughout the experimental period, resulting in a 100% survival rate across all treatments. Chickens reared under the OR system had significantly lower total FI, BWG, and final BW compared with those reared under the CO and FR systems (*P* < 0.05). No significant differences were found in FCR among the treatment groups. The results indicated that growth performance and feed intake varied with the different rearing conditions.

**Table 2:** The effect of different raising systems on growth performance of Korat broiler chickens at 91 days of age^1^.

Parameters	Production systems^2^			*SEM*	*P*-value
	CO	FR	OR		
Body weight (g)	1,502.60^a^	1,445.00^a^	1,173.80^b^	48.33	< 0.01
Body weight gain (g)	1,460.80^a^	1,403.00^a^	1,132.20^b^	27.06	< 0.01
Feed intake (g)	4,413.80^a^	3,861.00^ab^	3,307.60^b^	155.00	< 0.01
Feed conversion ratio (FCR)	3.06	2.76	2.91	0.06	0.16

a,b Means within a row with different superscript letters differ significantly at *P* < 0.05.

1 Values are means of 5 replicates (*n* = 75/treatment).

2 CO, Conventional system; FR, Free-range system; OR, Organic system. SEM, standard error of the mean.

Our findings for the OR group partially support the hypothesis that outdoor access may negatively affect chicken performance due to increased energy expenditure associated with foraging activity and environmental exposure. The significantly lower BWG and final BW in OR birds are consistent with the observations of Castellini et al. (2016), who indicated that reduced growth performance is likely attributable to the diversion of energy from growth toward physical activity and thermoregulation. Additionally, high pasture intake, as suggested by Singh and Cowieson (2013), may reduce the consumption of formulated diet, thereby negatively affecting energy balance and ultimately BW. However, this interpretation should be considered with caution, as pasture intake was not directly quantified in the present study.

Moreover, the results for the FR group were inconsistent with those reported by Wang et al. (2009), who observed reduced productivity in FR chickens. Despite having outdoor access, chickens in the FR group in the present study exhibited production performance comparable to that of the indoor-housed conventional group (CO). A possible explanation may relate to differences in outdoor space allowance between the FR and OR systems, which have been shown to influence chicken behavior and welfare (Dawkins et al., 2004). Li et al. (2017) also reported that pasture size can affect bird performance, while Forslind et al. (2021) and Evans et al. (2023) suggested that larger space availability may reduce competition and disturbances, thereby encouraging more resting behavior rather than foraging. Similarly, Zupan et al. (2003) examined the resting behavior in broilers reared under intensive, FR, and OR systems and observed that the percentage of birds resting outdoors was higher in the OR system (33.92%) than in the FR system (12.11%).

In the present study, the OR system provided a larger outdoor space allowance (4 m²/bird) than the FR system (1 m²/bird), which may have encouraged increased resting behavior. Collectively, these findings suggest that the interaction between housing environment and space allowance can influence chicken behavior and, consequently, productivity. However, further research is required to confirm these interpretations.

### The effect of rearing systems on observed behaviors and plumage damage

Behavioral observations have proven effective for assessing welfare-related issues in poultry (Nielsen et al., 2003; Dawkins et al., 2004). Rearing systems that restrict the expression of natural behaviors may increase welfare concerns, including emotional distress and the risk of feather pecking. In the present study, both the Kruskal-Wallis test and Principal Component Analysis (PCA) were applied to provide a comprehensive assessment of behavioral expression across the different rearing systems. As shown in Table 3, no statistically significant differences were observed in individual behavioral parameters among the three systems; however, distinct numerical trends were observed. Specifically, chickens in the OR and FR groups exhibited lower incidences of pecking behavior in the morning, as well as reduced frequencies of fighting and flapping wings in the afternoon, compared with those in the CO group (*P* = 0.06).

**Table 3:** The effect of different raising systems on observed behaviors of Korat broiler chickens at 91 days of age (Mean Rank)^1^.

Behaviors	Production systems^2^			Kruskal-Wallis H value^3^	*P*-value
	CO	FR	OR		
In Morning					
Pecking	8.00	4.00	3.00	5.60	0.06
Fighting	5.67	5.33	4.00	1.16	0.55
Drinking	4.83	5.00	5.17	0.22	0.98
Feeding	5.83	6.33	2.83	2.89	0.23
Preening	6.17	5.33	3.50	1.50	0.47
Dust bath	7.17	3.67	4.17	2.89	0.23
Foraging	5.50	5.67	3.38	0.82	0.66
Sitting	6.50	4.00	4.50	1.41	0.49
Flapping wings	5.83	5.17	4.00	0.70	0.70
Scratching	4.83	5.33	4.83	0.06	0.96
Shaking	5.83	5.67	3.50	1.41	0.49
Stretch	4.67	4.00	6.33	1.19	0.55
In Afternoon					
Pecking	7.67	4.00	3.33	4.35	0.11
Fighting	7.67	4.33	3.00	5.77	0.06
Drinking	3.00	5.00	7.00	3.20	0.20
Feeding	5.67	5.67	3.67	1.06	0.58
Preening	5.33	5.33	4.33	0.26	0.87
Dust bath	2.82	5.17	7.00	3.64	0.16
Foraging	2.67	6.33	6.00	3.28	0.19
Sitting	3.67	5.33	6.00	1.15	0.56
Flapping wings	8.00	4.00	3.00	5.69	0.06
Scratching	3.33	5.83	5.83	1.68	0.43
Shaking	5.33	5.33	4.33	0.26	0.87
Stretch	5.83	3.00	6.17	2.44	0.29

1 There are no significant differences in each row (*P* > 0.05).

2 CO, Conventional system; FR, Free-range system; OR, Organic system.

3 df = 2, *n* = 5.

**Fig. 2:**
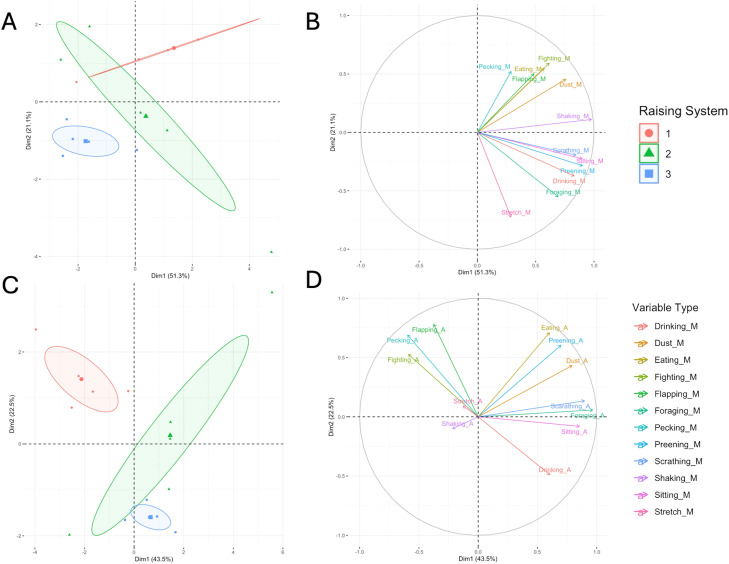
Principal component analysis (PCA) plots illustrating the relationship between rearing systems and behavioral patterns in slow-growing Korat broiler chickens. Each point represents a single replicate. Colors denote the experimental groups: conventional (1), free-range (2), and organic (3) systems. Score plot of the first two principal components (PC1 vs. PC2) showing distinct clustering of conventional (1), free-range (2), and organic (3) groups during the morning (A) and afternoon period (C). Loading plot identifying the specific behaviors driving the separation of the clusters during the morning (B) and afternoon period (D). The vectors indicate the direction and strength of each behavioral variable's contribution to the principal components.

Principal Component Analysis (PCA) was used to distinguish rearing systems based on observed chicken behaviors during both the morning and afternoon periods. For the morning period, the first two principal components (PC1 and PC2) explained 72.4% of the total variation, with PC1 accounting for 51.3% and PC2 for 21.1% (Figures 2A and 2B). Similarly, during the afternoon period, PC1 and PC2 together explained 66.0% of the total variance, contributing 43.5% and 22.5%, respectively (Fig. 2C and 2D). These results indicated that PCA effectively captured a substantial proportion of behavioral variation associated with the different rearing systems.

The PCA revealed that chickens reared under the CO system formed a distinct behavioral cluster compared with those in the FR and OR systems. Across both observation periods, CO birds exhibited a behavioral pattern characterized by higher frequencies of aggressive and active behaviors, including pecking, fighting, and flapping wings. These responses were likely exacerbated by the inherent temperament of the KRC, which retains genetic traits from Thai indigenous fighting cock lineages—breeds known for robust territorial instincts and social hierarchy-related aggression (Jaturasitha et al., 2002; Loengbudnark et al., 2024). While this aggression may be a potential trait of the breed, our results suggest it is significantly triggered by the environmental constraints of intensive housing, such as restricted space and higher stocking density (Dawkins et al., 2004).

In agreement with previous reports indicating that feather pecking is a major welfare concern, particularly in intensive production systems (Rodenburg et al., 2013). Elevated levels of aggressive activity have likewise been documented in confined chickens (Zepp et al., 2018; Ghayas et al., 2021). Increased aggression is strongly linked to elevated stress levels, which can lead to feather loss, skin injuries, and, in severe cases, cannibalism. These behavioral patterns could explain the poorer feather condition observed in CO chickens, particularly in the tail region, compared with FR and OR birds (*P* < 0.05; Table 4). This finding is in agreement with Sokołowicz et al. (2023), who reported superior head and tail feathering in laying hens reared under FR and OR systems compared with litter-based systems.

**Table 4:** The effect of different raising systems on plumage damage of Korat broiler chicken at 91 days of age (Mean Rank)^1^.

Area	Production systems^2^			Kruskal-Wallis H value^1^	*P*-value
	CO	FR	OR		
Back	27.26	23.00	24.70	2.07	0.35
Wings	28.38	25.15	21.00	5.16	0.07
Thigh	24.41	24.21	26.57	0.70	0.70
Tail	35.35^a^	19.50^b^	19.50^b^	25.65	< 0.01

a,b Means within a row with different superscript letters differ significantly at *P* < 0.05.

1 df = 2, *n* = 5.

2 CO, Conventional system; FR, Free-range system; OR, Organic system.

Feather loss in poultry is most commonly associated with pecking and aggressive interactions (Blatchford et al., 2016), with the back and tail regions often among the first areas to show visible plumage damage, indicating compromised welfare in both pecking recipients and initiators (Kjaer and Sørensen, 2002). Additional evidence suggests that access to outdoor ranging areas improves plumage condition and overall physical health (Nicol et al., 2013; Geng et al., 2023). Accordingly, the lower incidence of tail plumage damage observed in FR and OR chickens in the present study may be attributed to greater space availability and more diverse environmental conditions, which facilitate the expression of natural behaviors and reduce stress and feather pecking. This interpretation is supported by previous studies reporting that broilers with outdoor access display higher frequencies of natural behaviors, such as preening, dust bathing, and foraging, compared with birds housed indoors (Zhao et al., 2014). Moreover, FR and OR systems are generally considered more animal-friendly, as they provide increased space and environmental complexity that promote locomotor and exploratory behaviors, stimulated by external elements such as vegetation, soil, and food particles (Knierim, 2006; Dal Bosco et al., 2010, 2014).

Regarding feeding-related behaviors, PCA results indicated that chickens in both the CO and FR systems showed higher engagement in eating and drinking compared with those in the OR system. For the CO group, this finding is expected, as the restricted environment of confined housing encourages birds to rely primarily on indoor feeders and drinkers (Fiorilla et al., 2024). Interestingly, FR chickens also displayed more eating behavior during the afternoon period. Although access to pasture has been suggested to reduce dependence on indoor feed and water resources, this pattern was not observed in the present study. A possible explanation may relate to the high afternoon temperatures typical of tropical environments such as Thailand, which may discourage birds from ranging outdoors and instead promote indoor activity. Similar patterns have been reported by Dawkins et al. (2003), who noted that environmental factors, including season, ambient temperature, time of day, and resource availability, strongly influence ranging behavior. In addition, Dawkins et al. (2003) observed that birds tend to reduce outdoor activity during the hottest and brightest periods of the day. These behavioral responses may explain why FR chickens in this study showed feeding patterns comparable to those of CO birds.

Although birds with outdoor access are generally expected to display higher levels of walking and foraging rather than prolonged indoor behaviors such as eating, drinking, or lying, the OR chickens in the present study displayed a contrasting behavioral pattern. The PCA indicated that the OR system was associated with increased resting behaviors, particularly sitting and drinking. This observation is consistent with the findings of Zupan et al. (2003), who found that a greater proportion of chickens rested outdoors in the OR system compared with FR system, suggesting that birds in such environments may spend more time resting than engaging in active or exploratory behaviors.

Importantly, reduced activity levels in poultry have previously been associated with leg weakness, toe damage, or footpad dermatitis (Baracho et al., 2012); however, no mortality or clinical signs of disease were observed in the present study. Moreover, our previous work reported no significant differences in blood biochemical parameters or corticosterone concentrations among rearing systems (Khotsalee et al., 2024), suggesting that health impairment or physiological stress was unlikely to account for the observed behavioral pattern. Instead, a wide range of environmental, management, and bird-related factors has been reported to contribute to variability in ranging behavior (Campbell et al., 2025).

Chickens are known to preferentially use range areas that provide structural enrichment, such as trees, which offer shade, dry sites for dust bathing, and protection from aerial predators (Dawkins et al., 2003). In addition, both the density and structural quality of vegetation can influence the extent of outdoor use. Previous studies have demonstrated that enriched pasture environments can stimulate higher levels of locomotor activity and comfort behaviors, such as dust bathing and preening, compared with barren or indoor settings (Oke et al., 2021). In the present study, the availability of Ruzi grass in the FR and OR systems likely served as a functional form of environmental enrichment, encouraging natural behaviors that are otherwise restricted in the CO system.

Despite these insights, a significant knowledge gap remains regarding the precise behavioral thresholds of the KRC breed. It remains unclear whether the intensity of these aggressive interactions is driven primarily by the unique genetic makeup of indigenously derived crossbreds or the rearing environment itself. Consequently, future research should transition toward multi-factorial studies that integrate genetic profiling with precise metrics of range use. Such data will be vital for developing breed-specific welfare standards that balance the active nature of indigenous-derived chickens with the demands of sustainable poultry production.

### The effect of rearing systems on physical features of brain

Numerous studies have demonstrated that environmental complexity and enrichment can significantly affect brain morphology (Mehlhorn and Petow, 2020). Abnormal behaviors are often associated with neurobiological alterations, including brain injury, hippocampal neuron damage, dendritic remodeling in the hippocampus, or other forms of brain disorganization, which can reduce experiences of the positive welfare states in livestock animals (Campbell and Lee, 2021). Accordingly, the present study evaluated the effect of different rearing systems on the physical features of the brain.

As shown in Table 5, no significant difference was found in brain size among the groups. The similarity in brain size among groups may be explained by the fact that all birds were housed indoors until 21 days of age, a period during which major brain growth and organization are largely completed. Environmental stimulation is known to exert its greatest influence on neuronal formation during early life stages (Hübener and Bonhoeffer, 2014). Nevertheless, accumulating evidence indicates that neurogenesis and brain maturation continue into adulthood (Toda et al., 2019).

**Table 5:** The effect of different raising systems on brain structure^1^.

Parameters	Production systems^2^			*SEM*	*P-value*
	CO	FR	OR		
Brain weight (g)	3.65	3.64	3.59	0.48	0.75
Porosity (%)	27.09^a^	13.35^b^	8.71^b^	1.68	< 0.01
Volume (mm^3^)	19.50^b^	31.83^ab^	35.11^a^	2.72	0.04

a,b Means within a row with different superscript letters differ significantly at *P* < 0.05.

1 Values are means of 5 replicates (*n* = 20/treatment).

2 CO, Conventional system; FR, Free-range system; OR, Organic system. SEM, standard error of the mean.

Notably, chickens reared under the FR and OR systems exhibited a significantly lower percentage of brain porosity and a higher percentage of brain volume than those in the CO system (*P* < 0.01; Table 5). This finding suggests a divergence in neural tissue quality rather than overall mass, aligning with the principle that environmental complexity is vital for maintaining structural brain plasticity (Mehlhorn and Petow, 2020). These results are consistent with observations in laying hens, where outdoor access was associated with larger hippocampal neurons and enhanced serotonergic innervation (Patzke et al., 2009). Given that the avian hippocampus is a primary region for spatial orientation and memory (Tommasi et al., 2003), its proliferation has been shown to be highly responsive to ranging experiences (Armstrong et al., 2020), suggesting that extensive outdoor experience promotes a denser neural cytoarchitecture. Additionally, the diverse spatial challenges inherent in outdoor systems—such as estimating depth and distance—require continuous hippocampal engagement through its functional connection to the nidopallium caudolaterale region (**NCL**), a key area for avian visual cognition (Lee et al., 2012; Niu et al., 2022). As demonstrated by Freire and Cheng (2004), chickens exposed to visual barriers and complex spatial demands develop longer dendrites and increased spine density in the hippocampus. Such dendritic arborization effectively occupies more interstitial space within the brain tissue, providing a plausible biological explanation for the reduced porosity observed in our FR and OR groups. Conversely, the higher porosity in the CO system may indicate neural under-stimulation resulting from restricted spatial and cognitive demands. The observed differences in porosity and volume provide a potential neural basis for the varying degrees of cognitive adaptability and behavioral regulation reported across different housing systems. Notably, these micro-structural changes suggest that environmental enrichment promotes a denser neural architecture—a key component of neural plasticity and resilience in poultry.

However, a limitation of the present study is that brain assessment was restricted to whole-brain structural characteristics, without differentiation among specific brain regions. Given the areas such as the hippocampus, amygdala, and prefrontal cortex play critical roles in emotional regulation, learning, and stress adaptation (Patzke et al., 2009; Brunberg et al., 2011; Toda and Gage, 2018), future studies should investigate region-specific responses. In addition, molecular and neurochemical analyses are warranted to elucidate the mechanisms underlying stress-related neural adaptations. Such integrative approaches would provide deeper insight into how environmental enrichment shapes neural plasticity, brain resilience, and overall animal welfare in poultry production systems.

### The effect of rearing systems on breast meat quality and mineral content

Table 6 presents the effects of different rearing systems on breast meat quality and mineral content. Muscle glycogen content is closely related to the ultimate pH of meat. A lower ultimate pH is typically associated with higher glycolytic potential in muscle, which can result in increased drip loss and cooking loss, thereby negatively affecting chicken meat quality (Lesiów and Kijowski, 2003). Previous studies have reported that chickens raised outdoor access tend to exhibit lower muscle pH (Fanatico et al., 2006) and higher cooking loss (Castellini et al., 2002). These effects have been attributed to differences in pre-slaughter conditions and metabolic status, which may influence postmortem glycolysis and glycogen utilization. In contrast, the present study, together with the findings of Wang et al. (2009), Liu et al. (2011), and Tong et al. (2014), showed no significant effect of rearing system on muscle pH. This suggests that, under the conditions of the present study, differences in rearing systems may not have been sufficient to induce variations in pre-slaughter stress or muscle glycogen levels that would meaningfully affect ultimate pH.

**Table 6:** The effect of different raising systems on breast meat characteristics of Korat broiler chickens at 91 days of age^1^.

Parameters		Production systems^2^			*SEM*	*P*-value
		CO	FR	OR		
pH 45 min		5.93	5.84	5.85	0.01	0.07
Ultimate pH		5.93	5.87	5.84	0.02	0.18
Shear force (WBS)		2.70^b^	3.19^a^	3.18^a^	0.01	0.03
Drip loss (%)		8.56^a^	7.25^b^	7.14^b^	0.17	< 0.01
Cooking loss (%)		9.78	10.87	11.17	3.13	0.43
Collagen (mg/g)		0.44^b^	0.74^a^	0.79^a^	0.18	< 0.01
Skin color	Lightness	65.34	66.39	65.89	0.14	0.59
	Redness	−0.34	−0.81	−0.39	0.52	0.39
	Yellowness	5.28^b^	6.66^a^^b^	8.87^a^	1.62	0.03
Breast Meat color	Lightness	60.34	60.46	60.61	0.58	0.98
	Redness	−2.27	−2.18	−1.92	0.08	0.22
	Yellowness	2.17^b^	3.36^a^	4.36^a^	0.34	0.02
Iron (mg/kg)		2.28	2.64	2.08	0.36	0.44
Zinc (mg/kg)		3.61	3.83	3.48	0.08	0.22

a,b Means within a row with different superscript letters differ significantly at *P* < 0.05.

1 Values are means of 5 replicates (*n* = 20/treatment).

2 CO, Conventional system; FR, Free-range system; OR, Organic system. SEM, standard error of the mean.

Drip loss and cooking loss are important indicators of meat water-holding capacity **(WHC)**, representing the ability of muscle tissue to retain moisture during storage and cooking, respectively (Huff-Lonergan and Lonergan, 2005). Higher drip loss reflects greater exudation of water-soluble nutrients and flavor compounds, thereby reducing meat quality (Liu et al., 2011). Although cooking loss of breast meat did not differ significantly among the three rearing systems, our results indicated that meat from FR and OR chickens exhibited significantly lower drip loss compared with that from CO chickens (*P* < 0.05). This finding agrees with Yang et al. (2015), who reported lower drip loss in meat from free-range chickens than in meat from birds in indoor floor systems.

The improved WHC in FR and OR meat may be attributed to the enhanced structural integrity of the intramuscular connective tissue. Higher physical activity in outdoor ranges stimulates the synthesis of a more robust collagen matrix (Lin et al., 2014). This strengthened framework provides superior structural support to the myofibrillar lattice, effectively stabilizing the muscle architecture and limiting the shrinkage that leads to water exudation during post-mortem storage (Purslow, 2018).

However, this structural adaptation appears to be linked to a trade-off in meat tenderness—a key factor in consumer acceptance (Weston et al., 2002). In the present study, breast meat from FR and OR chickens exhibited higher collagen content and shear force values compared to the CO group (*P* < 0.05), suggesting that increased shear force may be a consequence of elevated protein and collagen concentrations within the muscle tissue. Beyond total concentration, locomotor activity is known to influence the maturation of the intramuscular connective tissue; specifically, increased muscle use promotes the formation of mature, heat-stable cross-links within the collagen fibers (Wang et al., 2023; Zhang et al., 2026). These cross-links enhance the thermal stability of the connective tissue, making it less soluble during heat treatment and resulting in the firmer texture and higher shear force observed (Fletcher, 2002). Collectively, these findings suggest that while extensive rearing improves water retention, possibly through a reinforced collagen matrix, it simultaneously produces a more robust, less tender meat texture, likely as a physiological adaptation to increased muscle activity.

Beyond tenderness, skin and meat color are important sensory attributes that strongly influence consumer preference, especially in natural and organic poultry markets (Fletcher, 2002). Although variations in lightness (L*) and redness (a*) generally have limited practical implications for poultry meat quality (Funaro et al., 2014), chickens reared under the OR group exhibited the highest yellowness (b*) values in both meat and skin. Similar findings have been reported by Fanatico et al. (2006) and Molee et al. (2022), who observed that birds with outdoor access tend to display more pronounced yellow pigmentation. This effect is commonly attributed to the ingestion of carotenoid-rich plant materials during foraging, which enhances pigment deposition in skin and muscle tissues (Chen et al., 2013).

Regarding mineral composition, it was hypothesized that the FR and OR groups would exhibit higher concentrations of iron and zinc due to increased physical activity and the potential intake of minerals from stone and forage in extensive systems. In meat science, these minerals are critical; iron is a core component of myoglobin—the primary pigment responsible for meat color—and its levels typically rise with increased muscle oxidative metabolism (Husak et al., 2008). Similarly, zinc serves as an essential cofactor for various antioxidant enzymes that help maintain postmortem oxidative stability (De Grande et al., 2022; Xiao et al., 2025). Contrary to our initial hypothesis, however, our results showed no significant differences in the iron or zinc contents of breast meat among the three rearing systems. This stability in iron content is reflected in the meat's visual characteristics; since iron is a primary component of myoglobin, its uniform concentration across groups likely explains why no significant variations in redness were detected. These results partially contrast with those of Lin et al. (2014), who reported that while production systems had no effect on zinc levels, they significantly increased iron content in chickens with outdoor access. The absence of significant differences in the present study, despite the increased locomotor activity in the FR and OR systems, may reflect variations in genotype, age at slaughter, physiological status, or a relatively low mineral density in the specific outdoor range used. Nevertheless, the consistent levels across groups ensure that the nutritional quality of KRC meat remains stable regardless of the rearing system used.

### The effect of rearing systems on fatty acid composition

Table 7 presents the effects of different rearing systems on the fatty acid (FA) composition of chicken breast muscles. Linoleic acid (**LA**; C18:2n-6) was the predominant FA, followed by palmitic acid (**PA**; C16:0), dihomo-γ-linolenic acid (**DGLA**; C20:3n-6), oleic acid (**OLA**; C18:1n-9), and stearic acid (**SA**; C18:0). No significant differences were observed in the total contents of saturated fatty acids (**SFA**), monounsaturated fatty acids (**MUFA**), or polyunsaturated fatty acids (**PUFA**) among the rearing systems. However, docosahexaenoic acid (**DHA**; C22:6n-3) content was significantly higher in chickens reared under the FR and OR systems compared with the CO system (*P* < 0.05). In addition, free-range and organically raised chickens exhibited the lowest n-6/n-3 PUFA ratio (*P* < 0.05) compared with the CO group, indicating a more favorable FA profile in terms of nutritional value.

**Table 7:** Effect of different rearing systems on fatty acid composition in Korat broiler chickens at 91 days of age^1^.

Fatty acid (g/100 g total FA)	Production systems^2^			*SEM*	*P*-value
	CO	FR	OR		
C14:0	0.37	0.30	0.37	0.03	0.56
C16:0	23.75	23.93	23.82	0.85	0.17
C16:1	0.57	0.31	0.49	0.06	0.11
C18:0	11.33	11.44	11.81	0.46	0.81
C18:1n-9	17.71	18.62	18.35	1.43	0.45
C18:2n-6	24.74	23.77	24.15	1.13	0.29
C18:3n-6	0.08	0.07	0.07	< 0.01	0.52
C18:3n-3	0.76	0.79	0.76	0.08	0.95
C20:0	0.13	0.09	0.13	0.01	0.40
C20:2	0.69	0.66	0.64	0.01	0.46
C20:3n-6	0.45	0.66	0.53	0.05	0.23
C20:4n-6	16.98	15.82	15.02	1.38	0.21
C20:5n-3	0.22	0.30	0.23	0.02	0.32
C22:6n3	2.22^b^	3.24^a^	3.63^a^	0.19	0.05
SFA	41.78	36.34	41.04	1.36	0.24
MUFA	26.07	22.14	25.56	1.30	0.47
PUFA	32.15	41.62	33.40	2.46	0.29
Total n-6	38.66	39.04	42.36	2.46	0.29
Total n-3	3.74^b^	4.47^a^^b^	4.89^a^	0.19	0.03
n-6/n-3	10.38^a^	8.77^b^	8.66^b^	0.29	< 0.01

a,b Means within a row with different superscript letters differ significantly at *P* < 0.05.

1 Values are means of 5 replicates (*n* = 20/treatment).

2 CO = Control, FR = Free-range system, OR = Organic system. SEM, standard error of the mean.

Previous studies have demonstrated that both dietary fat composition and rearing system influence the fatty acid profile of poultry meat (Husak et al., 2008). Providing chickens with access to pasture, where they can express natural behaviors and ingest additional nutrients such as vitamins, minerals, and antioxidants, has been shown to enhance meat nutritional quality, particularly by increasing PUFA content (Lichovníková et al., 2009; Hoan and Khoa, 2016). Consistently, Dal Bosco et al. (2012) and Molee et al. (2022) reported that slow-growing birds with outdoor access engaged more actively in foraging and consumed green forage rich in PUFA, especially n-3 PUFA.

Fresh grass, containing approximately 50–75% α-linolenic acid (**ALA**), serves as a key precursor for long-chain n-3 PUFA synthesis. Through elongation and desaturation processes, ALA can be converted into eicosapentaenoic acid (**EPA**; C20:5n-3), docosapentaenoic acid (**DPA**; C22:5n-3), and DHA (Gálvez et al., 2020), which likely contributed to the elevated DHA levels observed in meat from the FR and OR groups.

Furthermore, a low n-6/n-3 PUFA ratio is well recognized as beneficial for human health (Bishehkolaei and Pathak, 2024). In this study, meat from FR and OR chickens exhibited the lowest n-6/n-3 PUFA ratio among the groups, consistent with the findings of Michalczuk et al. (2017). This reduction is likely due to the higher n-3 PUFA content derived from greater access to ALA-rich forage. The competition among fatty acids for desaturase and elongase enzymes may further explain this trend, as these enzymes preferentially convert n-3 over n-6 fatty acids when substrate availability is favorable (Cortinas et al., 2004).

However, the absence of a significant difference in the n-6/n-3 ratio between the FR and other groups suggests that outdoor foraging alone may not strongly influence fatty acid composition. Differences in forage availability, nutrient composition, and actual forage intake across systems may explain this variability. Ponte et al. (2008) highlighted that the nutritional quality and accessibility of forage can greatly affect the fatty acid profile of poultry meat, though outcomes may vary across systems. Moreover, non-dietary factors, such as increased physical activity or social stress associated with higher stocking density, can induce oxidative stress, thereby altering fatty acid stability and composition (Dal Bosco et al., 2012; Zepp et al., 2018). Thus, further research is warranted to elucidate how different rearing systems influence lipid metabolism and oxidative stability in poultry meat, thereby optimizing production strategies to improve nutritional quality.

## Conclusion

This study provides novel insights into how rearing environments influence brain structural integrity, behavioral expression, and meat quality in slow-growing chickens. Extensive systems (FR and OR) reduced growth rates but promoted behavioral homeostasis and neuro-structural development, mitigating aggression associated with intensive housing. Environmental complexity also improved key meat traits, including water-holding capacity, n-3 PUFA content, and skin pigmentation. These findings provide a scientific basis for welfare-oriented management, enabling producers to enhance animal welfare and meat quality while offsetting slower growth through improved product characteristics.

## CRediT authorship contribution statement

**Pramin Kaewsatuan:** Writing – review & editing, Writing – original draft, Visualization, Validation, Formal analysis, Data curation. **Wichuta Khosinklang:** Visualization, Validation, Investigation, Data curation. **Sirirot Khotsalee:** Investigation, Formal analysis, Data curation. **Catleya Rojviriya:** Supervision, Data curation. **Wunpen Sonsamrong:** Data curation, Formal analysis, Investigation. **Mutyarsih Oryza:** Visualization, Validation, Investigation, Formal analysis. **Amonrat Molee:** Visualization, Supervision, Methodology, Conceptualization. **Wittawat Molee:** Writing – review & editing, Supervision, Conceptualization.

## Disclosures

The authors declare that they have no known competing financial interests or personal relationships that could have appeared to influence the work reported in this paper.
